# *Fgf8/18* antagonizes *Shh* expression in lingual ventral–dorsal patterning

**DOI:** 10.3389/fcell.2026.1724475

**Published:** 2026-02-10

**Authors:** Shuhui Yang, Junyuan Xue, Han Liu, Nan Zhou, Hui Feng, Nan Li, Bo Liu, Lei Zhu, Jing Xiao, Chao Liu

**Affiliations:** 1 Department of Oral Pathology, School of Stomatology, Dalian Medical University, Dalian, China; 2 Academician Laboratory of Immunology and Oral Development & Regeneration, Dalian Medical University, Dalian, China; 3 Institute for Genome Engineered Animal Models of Human Diseases, Dalian Medical University, Dalian, China

**Keywords:** dorsal–ventral pattern, Fgf18, Fgf8, lingual deformity, sonic hedgehog, tongue development

## Abstract

**Introduction:**

The cellular and molecular mechanisms in tongue development are still poorly understood. Explicating how the developing tongue is patterned into a dorsally wide and ventrally narrow asymmetry would benefit the pathological interpretation of tongue deformities.

**Methods:**

In this study, we first revealed that the dorsal extension of *Fgf8* from the ventral mesenchyme in *Osr2-cre* ^*KI*^ *;Rosa26R-Fgf8* mouse embryonic tongues disrupted dorsal–ventral asymmetry by suppressing the cell proliferation and tenogenic differentiation of lingual dorsal mesenchyme. By intersecting the differentially expressed genes (DEGs) in mouse embryonic dorsal tongues with the canonical gene set of dorsal–ventral pattern formation, *Shh* and *Shh*-related genes were found to be specifically activated in the embryonic dorsal tongue. The DEGs between WT dorsal and *Osr2-cre* ^*KI*^ *;Rosa26R-Fgf8* dorsal tongues showed that the expression of *Lhx6*, an *Fgf8/18*-related transcription factor robustly expressed in the WT ventral tongue, was increased in the *Osr2-cre* ^*KI*^ *;Rosa26R-Fgf8* dorsal tongue.

**Results:**

Histological assays verified that in both *Osr2-cre* ^*KI*^ *;Rosa26R-Fgf8* and *Shh-cre;Rosa26R-Fgf8* embryonic tongues, the expression of *Shh* and *Shh*-related genes, including *goosecoid* (*Gsc*), *Foxa2*, and *Foxf1*, was suppressed in the dorsal area, while the transcription of the ventrally located *Fgf8/18*-related *Lhx6* was extended into the dorsal area. FGF8 or FGF18 supplementation in WT tongues recapitulated the suppression of *Shh* and *Shh*-related genes. However, exogenous SHH neither suppressed *Fgf18* and *Lhx6* nor activated the *Shh*-related gene *Foxf1* in the lingual ventral mesenchyme. These results indicate the involvement of *Shh* and *Fgf8/18* in lingual dorsal–ventral patterning, in which ventral *Fgf8/18* suppresses the extension of dorsal *Shh.*

**Discussion:**

Our findings not only confirm the existence of dorsal–ventral patterning during tongue development but also identify *Shh* and *Fgf8/18* as key genes defining the lingual dorsal–ventral axis, providing cellular and molecular clues for interpreting the clinical manifestations of congenital lingual deformities.

## Introduction

Clinical practice has identified multiple congenital tongue malformations, such as aglossia, microglossia, bifid tongue, and ankyloglossia, that impair chewing, swallowing, and speaking (Cobourne et al., 2019). However, the etiology and pathogenesis of these malformations remain poorly understood because the cellular and molecular mechanisms involved in tongue development are still elusive.

*Shh* expressed in the lingual dorsal epithelium is critical for tongue development. At embryonic day (E) 10.5 of mouse gestation, *Shh* is activated in the distal oral epithelium of the mandibular arch and stimulates the proliferation of the underlying cranial neural crest-derived mesenchymal cells (CNCCs) to form two lateral lingual swellings that emerge into the lingual primordium at E11.5 (Jung et al., 1999). Neutralizing SHH with antibodies, genetic ablation of ectodermal *Shh*, or inactivation of the SHH signaling mediator *Smoothened* (*Smo*), *Gli2* and *Gli3*, or the transducer *Kif3a* in CNCCs results in aglossia by disrupting lingual genesis (Liu et al., 2004; Billmyre and Klingensmith, 2015; Millington et al., 2017). With the ventral and proximal extension of *Shh* expression in E11.5 lingual epithelium, the lingual mesenchyme activates *Wnt5a* in the distal and CXCL12 in the proximal, through which the CXCR4+ myogenic progenitors in the hypoglossal cord are induced to invade into the lingual primordium. Inactivating *Shh* expression by deleting *Islet1* in the lingual epithelium abrogates *Wnt5a* transcription in the lingual distal mesenchyme, which blocks the proximal CXCL12+ mesenchyme and CXCR4+ myoblasts out of the lingual primordium (Zhang et al., 2022). At E12.5, although *Shh* expression is restricted to fungiform papillae on the lingual dorsal epithelium, SHH signaling is still active in the underlying mesenchyme (Jung et al., 1999) to pattern the lingual tendon formation. Attenuated SHH signaling in lingual mesenchyme impairs the formation of the lingual septum and tendons, which, in turn, interrupts myofiber arrangement and intrinsic lingual muscle patterning (Okuhara et al., 2019). The targets of SHH signaling, *Foxf1* and *Foxf2*, are indicated to regulate the tenogenic differentiation of lingual mesenchyme, along with myoblast differentiation and fusion, by activating *Hgf*, *Tgfb2*, and *Tgfb3* in lingual mesenchyme (Iwata et al., 2013; Xu et al., 2022). A recent study demonstrated that SHH signaling in CNCCs maintains the myogenic fate of the migrating myogenic progenitors by mediating the interactions between CNCCs and myogenic cells (Kawasaki et al., 2024).

Anatomically, the lingual intrinsic muscles exhibit a medial–lateral patterning where the vertical and transverse groups attached to the central septum are completely encircled by the superior and inferior longitudinal muscles (Parada et al., 2012). The tongue also shows a dorsal–ventral pattern with dorsal–ventral asymmetry, especially in the distal end, where the dorsal portion is wider than the ventral portion. Additionally, both *Foxf1* and *Foxf2* exhibit a dorsal-specific expression pattern in the lingual mesenchyme. Double knockout of *Foxf1* and *Foxf2* in CNC-derived mesenchyme narrows the tongue dorsally (Xu et al., 2022), implicating a disrupted dorsal–ventral patterning. Although specifically expressed in the dorsal lingual epithelium, the dorsalizing role of *Shh* in tongue development remains unknown. Moreover, the signal ventralizing lingual mesenchyme also requires elucidation.

The expression patterns of multiple *Fgfs* in a developing tongue have already been reported (Du et al., 2016). *Fgf10* is activated in the dorsal mesenchyme to promote cell proliferation (Hosokawa et al., 2010; Song et al., 2013), and FGF6 released from myoblasts enhances myoblast fusion (Han et al., 2012). In the lateral lingual swellings, *Fgf8* is first detected in the mesenchyme underlying the *Shh*-expressing epithelium, and its expression decreases at E12.5 with increasing *Shh* transcription (Jung et al., 1999). On the other hand, *Fgf18*, an ortholog of *Fgf8* in the *Fgf8* subfamily (Hao et al., 2019), is robustly expressed in the lateral ventral mesenchyme at E12.5 (Du et al., 2016). A previous study reported that deletion of *Foxf2* ectopically activated *Fgf18* in the palatal mesenchyme but diminished *Shh* in the palatal epithelium. Consistently, FGF18 supplementation on palatal shelves represses *Shh* transcription (Xu et al., 2016). Thus, we hypothesize that in the early lingual primordium, FGF8/18 from the ventral mesenchyme antagonizes SHH from the dorsal epithelium to define dorsal–ventral patterning.

In this study, we extend *Fgf8* expression from the lateral ventral mesenchyme to the dorsal mesenchyme of the tongue using the *Osr2-cre* knock-in allele. The *Osr2-cre* ^*KI*^ *;Rosa26R-Fgf8* mouse embryos displayed an oval tongue without dorsal–ventral asymmetry, along with reduced *Shh* transcription in the dorsal epithelium and suppressed proliferation and tenogenic differentiation in the dorsal mesenchyme. Our study further revealed that in both *Osr2-cre* ^*KI*^ *;Rosa26R-Fgf8* and *Shh-cre;Rosa26R-Fgf8* tongues, the ventral-specific transcription factor *Lhx6* extended dorsally, while the dorsal-specific transcription factors, *Gsc*, *Foxa2*, and *Foxf1/2*, were limited in a much narrower dorsal area, suggesting that the suppression of dorsal *Shh* and *Shh*-related genes by ventral FGF8/18 defines the dorsal–ventral patterning during tongue development.

## Materials and methods

### Mouse lines

All *Osr2-cre* ^*KI*^, *Shh-cre*, *Rosa26R-mT/mG*, and *Rosa26R-Fgf8* lines were maintained under specific pathogen-free conditions at the Institute of Genome Engineered Animal Models for Human Diseases, Dalian Medical University, and were identified by genotyping PCR, as described previously (Liu et al., 2022; Huang et al., 2023). All animal procedures were conducted in accordance with the “Guide for the Care and Use of Laboratory Animals” (National Institutes of Health) and a protocol approved by the Animal Care and Use Committee of Dalian Medical University (Protocol No. AEE18011).

### Histology and cryostat section

The timed-pregnant mice were euthanized with carbon dioxide, followed by cervical dislocation. For histology, embryonic mouse heads were fixed in 4% paraformaldehyde overnight, dehydrated through graded alcohols, embedded in paraffin, and sectioned at 10 µm thickness for Masson staining. For cryostat section, more than five *Osr2-cre* ^*KI*^ *;Rosa26R-mT/mG* and *Shh-cre;Rosa26R-mT/mG* embryonic mouse heads were fixed in a mixture of 4% paraformaldehyde and 15% sucrose overnight, dehydrated in 30% sucrose overnight, embedded in O.C.T. compound, and sectioned at 10 µm thickness. To measure the tongue length, three mandibles with tongues were dissected from E16.5 embryonic heads prior to fixation.

### Bulk RNA-seq analysis

The lingual tissue from the distal tip to the frenum was defined as the anterior tongue that was dissected from the posterior section by sharp blades. The horizontal line passing through the mid-point of the vertical median line of the tongue was applied to delineate the boundary separating the dorsal and ventral tongue. The E13.5 *WT* and *Osr2-cre;Rosa26R-Fgf8* tongues were cut off from the frenum, and the anterior parts were further dissected into dorsal and ventral portions. Three *Osr2-cre* ^*KI*^ *;Rosa26R-Fgf8* embryos and their WT littermates in each litter were collected for bulk-RNA-seq, and three different litters were repeated. Following total RNA extraction with TRIzol reagent (Invitrogen), RNA quality was assessed using the 2100 Bioanalyzer System (Agilent) and quantified using the ND-2000 (NanoDrop Technologies). RNA-seq libraries were constructed using the TruSeq RNA Sample Preparation Kit (Illumina, San Diego, CA) and sequenced on the Illumina NovaSeq 6000 platform. Reads were aligned to the GRCm39 genome using HISAT2 with default parameters. Aligned data quality was assessed by Qualimap (Okonechnikov et al., 2016), and HTSeq (Anders et al., 2015) was used to count gene-mapped reads. The DESeq2 (Love et al., 2014) package was used to analyze differentially expressed genes (DEGs) based on read counts. The dorsal–ventral patterning gene set was downloaded from the Molecular Signatures Database (MSigDB) (via https://www.gsea-msigdb.org/gsea/msigdb/mouse/geneset/GOBP_DORSAL_VENTRAL_PATTERN_FORMATION.html?keywords = dorsal) with a version history note: 2025.1.Mm: Updated to GO Release 2025-03-16. The canonical pathway enrichment analysis was conducted using the enricher function in the clusterProfiler (Yu et al., 2012) R package with gene sets sourced from MSigDB M2 CP. The selected genes were imported into the STRING database to construct a protein–protein interaction (PPI) network, and Cytoscape, with its CytoCluster plugins, was used for network visualization and subnetwork extraction to explore gene interaction patterns. Gene expression data of each sample were mapped to the dorsal tongue gene set, and their activity levels were calculated using the GSVA (Hänzelmann et al., 2013) R package.

### Whole-mount *in situ* hybridization and immunofluorescence staining

Whole-mount *in situ* hybridization with the *Shh* anti-sense RNA probe was performed according to the previous procedure (Chen et al., 2023). At least three replicates were performed to ensure the consistency of *Shh* expression. Immunofluorescence staining was performed as described previously (Chen et al., 2025). The primary antibodies included the antibodies against Ki67 (1:2000; Abcam, ab15580), myosin (1:60000; Abcam, ab37484), Scx (1:150; Abcam, ab307722), MyoD (1:2000; Thermo Fisher, MA1-41017), cytokeratin 8 (1:2000; Abcam, ab53280), Foxf1 (1:2000; Abcam, ab308633), Foxa2 (1:2000; Abcam, ab256493), Gsc (1:300; Abbexa, abx326880), Lhx6 (1:800; Abcam, ab300441), and Etv4 (1:2000; Proteintech, 10684-1-AP). The secondary antibody was included in the MaxVision^TM^ HRP Polymer anti-Mouse/Rabbit IHC Kit (No. KIT5020, Maixin Ltd., Fuzhou, China). The slices were incubated with iF Tyramide (1:1000; ServiceBio, G1236-50T) for 10 min and counterstained with DAPI (Solarbio, S2100). For each assay, the *Shh-cre;Rosa26R-Fgf8* or *Osr2-cre* ^*KI*^ *;Rosa26R-Fgf8* embryos and their WT littermates from at least three different litters were repeated. The two-tailed Student’s t-test for Ki67 percentages was performed using GraphPad Prism software (version 8.0.1), and the results are presented as the means ± standard deviation, with statistical significance set at *p* < 0.05.

### Organ culture

The agarose beads (Bio-Rad, 1537302) were incubated in phosphate-buffered saline containing 0.5 μg/mL of FGF8 (Cat. 100-25-2 µg; Thermo Fisher), FGF18 (Cat. 100-28-5 µg; Thermo Fisher), or SHH (HY-P7290; Mce) for 1 hour and then grafted into tongues dissected from E13.0 mouse embryos. After 12 h of organ culture in Trowell dishes, the tongues were fixed overnight in 4% paraformaldehyde for immunofluorescence staining or whole-mount *in situ* hybridization. For each growth factor, the implantation was repeated thrice in at least three different litters. Three replicates were performed for FGF8-, FGF18-, and SHH-soaked bead implantation, respectively.

### Statistical analysis

Ki67-positive cells and the total number of cells within the restricted areas were counted using the counting tool in Photoshop, data analysis was performed using a two-tailed Student’s t-test, statistical significance was set at *p* < 0.05, and quantitative data were all shown as the means ± standard deviation, based on three independent replications. For comparative analyses on the bulk RNA-seq of the dorsal and ventral tongues of E13.5 WT and *Osr2-cre* ^*KI*^ *;Rosa26R-Fgf8* mice, one-way ANOVA and Tukey’s multiple comparisons test were used to identify the intergroup differences, and significance was set at adj.P < 0.05. For the quantitative measurement of *Shh* transcription by whole-mount *in situ* hybridization and signal intensity in immunofluorescence, ImageJ (version 1.54 g) was used to quantify color or fluorescence intensity and the percentage of signal areas. For bead-implanted whole-mount *in situ* hybridization and immunofluorescence assays, the color or fluorescence intensity in the surrounding areas of the beads was quantified. The surrounding areas of the beads were defined as the area around a bead with a fixed distance. The images were converted to 8-bit grayscale, and the mean gray values were measured for whole-mount *in situ* hybridization, whereas the mean fluorescence intensity was measured for the immunofluorescence assay. The background color/fluorescence measured from the adjacent unstained or fluorescence-free regions was subtracted to obtain the corrected mean color/fluorescence intensity. For immunofluorescence assays without bead implantation, the fluorescence intensity was quantified after grayscale conversion. A constant threshold was applied across all samples to identify the signal-positive area, which was expressed as a percentage of the total lingual area. All statistical analyses were conducted using GraphPad Prism software (version 8.0.1).

## Results

### Attenuated dorsal features in the *Osr2-Cre* ^*KI*^ *;Rosa26R-Fgf8* tongue

**FIGURE 1:**
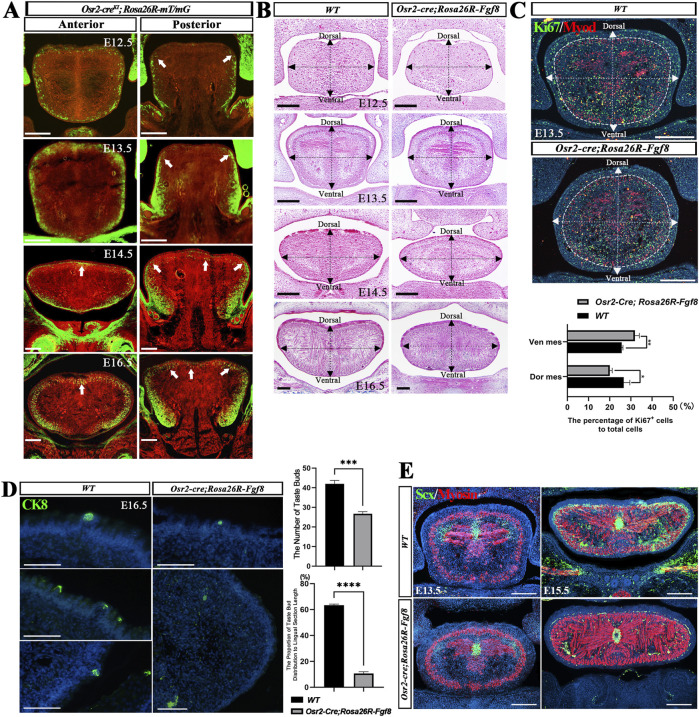
Missing dorsal–ventral asymmetry and dorsal features in the tongues of *Osr2-cre* ^*KI*^ *;Rosa26R-Fgf8* mouse embryos. **(A)** *Osr2-cre* ^*KI*^ *;Rosa26R-mT/mG* tongues showed that *Osr2-Cre* was activated in both the dorsal and ventral superficial mesenchyme from E12.5 to E15.5 but expanded into the mesenchyme surrounding the inferior and superior longitudinal muscles at E16.5. White arrows indicate the sporadic initial activation of *Osr2-cre* ^*KI*^ in the dorsal mesenchyme underlying the lingual mucosa. **(B)** Cross-sections of the developing *Osr2-cre* ^*KI*^ *;Rosa26R-Fgf8* tongues that did not show dorsal–ventral asymmetry. The vertical dashed dotted line is the median line dividing the tongue into the right and left parts, while the horizontal dashed dotted line passes through the mid-point of the vertical dashed dotted line to divide the tongue into the dorsal and ventral parts. **(C)** Statistical assay of the immunofluorescence of Ki67 and MyoD showed that compared to that in the WT controls, cell proliferation was increased in the ventral superficial mesenchyme (WT: 25.69% ± 0.57% vs. *Osr2-cre* ^*KI*^ *;Rosa26R-Fgf8*: 31.96% ± 2.02%, **: *p* < 0.01) but decreased in the dorsal superficial mesenchyme (WT: 26.65% ± 2.86% vs. *Osr2-cre* ^*KI*^ *;Rosa26R-Fgf8*: 20.07 %± 1.13%, *: *p* < 0.05) in E13.5 *Osr2-cre* ^*KI*^ *;Rosa26R-Fgf8* tongues. The dashed white circles delineate the boundary between the superficial and medial mesenchyme; the vertical dashed dotted line is the median line separating the tongue into the right and left parts, while the horizontal dashed dotted line passes through the mid-point of the vertical dashed dotted line to divide the tongue into the dorsal and ventral parts. **(D)** Immunofluorescence of CK8 showed fewer and dorsally distributed taste buds in E16.5 *Osr2-cre* ^*KI*^ *;Rosa26R-Fgf8* tongues compared to WT controls. The number of CK8+ taste buds in the WT tongue (42.81 ± 1.73/tongue) was significantly higher than that in *Osr2-cre* ^*KI*^ *;Rosa26R-Fgf8* tongues (26.67 ± 1.16/tongue, ***: *p* < 0.001). Moreover, the distribution of CK8+ taste buds occupied a lower proportion in the *Osr2-cre* ^*KI*^ *;Rosa26R-Fgf8* lingual epithelium (10.68% ± 1.34%/tongue) compared to the WT lingual epithelium (63.45% ± 0.81%/tongue, ****: *p* < 0.0001). **(E)** Immunofluorescence of Scx and myosin showed that in E13.5 *Osr2-cre* ^*KI*^ *;Rosa26R-Fgf8* tongues, *Scx* expression was suppressed in the lateral–dorsal mesenchyme but not in the central septum; in E16.5 *Osr2-cre* ^*KI*^ *;Rosa26R-Fgf8* tongues, *Scx* expression in both the superior and inferior muscles was attenuated, while it was still robust in the central septum. Scale bar: 200 µm.

We previously showed that *Osr2-Cre* ^*KI*^ *;Rosa26R-Fgf8* mice displayed micrognathia (Liu et al., 2022), which caused the tongue to protrude from the oral cavity as the length of the *Osr2-Cre* ^*KI*^ *;Rosa26R-Fgf8* tongue was comparable to that of the WT control (Supplementary Figures S1A, B). Our analysis of the *Osr2-cre* ^*KI*^ expression pattern through the *Rosa26R-mT/mG* reporter line indicated that in the anterior tongue, *Osr2-cre* ^*KI*^ was activated throughout the superficial mesenchyme underlying epithelium at E12.5 and extended into the mesenchyme surrounding longitudinal muscles from E14.5 to E16.5 (Figure 1A). However, in the posterior region, *Osr2-cre* ^*KI*^ expression was restricted in the bilateral superficial mesenchyme at E12.5, extended into the mesenchyme surrounding longitudinal muscles from E13.5, and converged dorsally at E16.5 (Figure 1A). From E12.5 to E16.5, *Osr2-Cre* ^*KI*^ *;Rosa26R-Fgf8* tongues exhibited a symmetrically oval shape in the anterior region instead of the dorsally wide–ventrally narrow asymmetry observed in WT control (Figure 1B). Further histology revealed that the posterior tongue of *Osr2-Cre* ^*KI*^ *;Rosa26R-Fgf8* mice was severely deformed by the compression from the enlarged palatal shelves (Supplementary Figure S1C), while the anterior tongue was not in contact with the palatal shelves (Figure 1B). Thus, we focused on the anterior tongue to explore the disrupted dorsal–ventral patterning in *Osr2-Cre* ^*KI*^ *;Rosa26R-Fgf8* mice. Ki67 staining indicated that compared to the WT counterparts, the proliferation of superficial mesenchymal cells was increased in the ventral but decreased in the E13.5 *Osr2-Cre* ^*KI*^ *;Rosa26R-Fgf8* dorsal tongue (Figure 1C). In contrast, the TUNEL assay indicated comparable cell apoptosis in both E13.5 WT and *Osr2-Cre* ^*KI*^ *;Rosa26R-Fgf8* tongues (Supplementary Figure S1D). Additionally, in contrast to the wide distribution in the WT dorsal and lateral lingual epithelium, CK8+ taste buds were only detected in the central area of *Osr2-Cre* ^*KI*^ *;Rosa26R-Fgf8* dorsal lingual epithelium (Figure 1D). Moreover, although it persisted in the septum, *Scx* expression was absent in the E13.5 dorsal lingual mesenchyme and was fainter in the E15.5 superficial mesenchyme of *Osr2-Cre* ^*KI*^ *;Rosa26R-Fgf8* tongues (Figure 1E). Taken together, all these attenuated dorsal features indicated a disrupted dorsal–ventral patterning in *Osr2-Cre* ^*KI*^ *;Rosa26R-Fgf8* tongue.

### Suppressed *Shh*-related dorsal-specific genes in the *Osr2-Cre* ^*KI*^ *;Rosa26R-Fgf8* tongue

**FIGURE 2:**
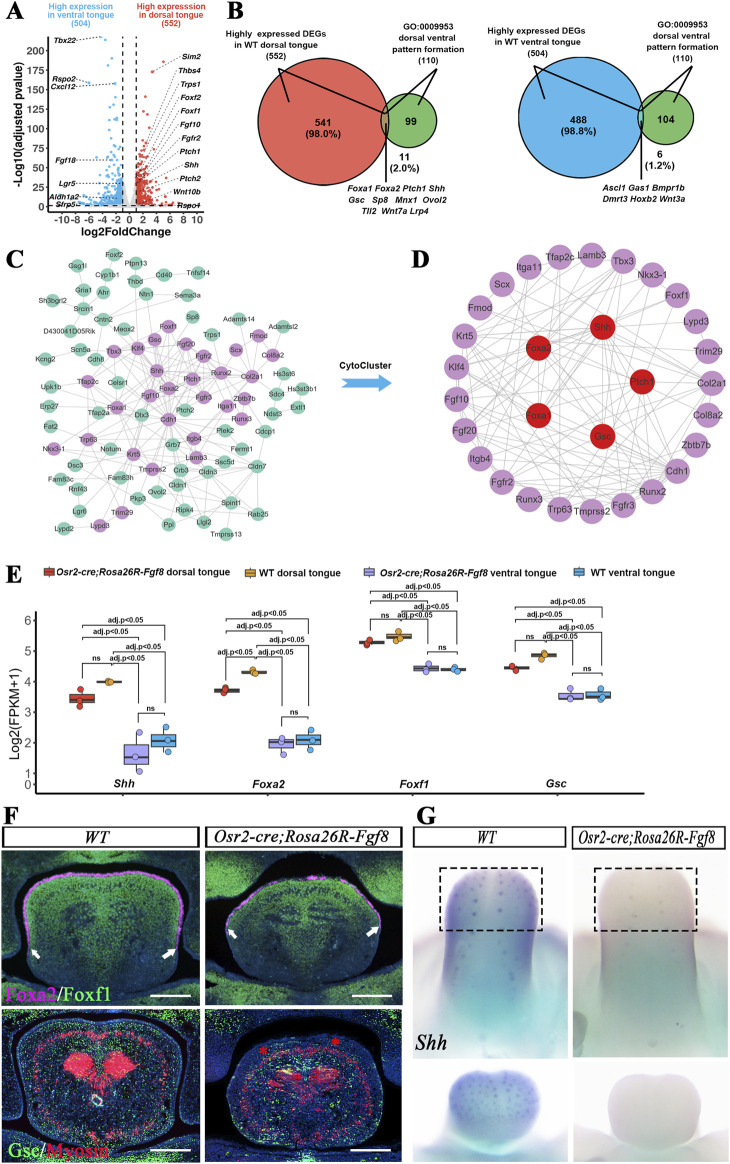
Bulk RNA-seq analysis associated the *Shh*-related genes with lingual dorsal–ventral patterning. **(A)** Volcano plot displaying the DEGs between E13.5 WT dorsal and ventral tongues. **(B)** Venn diagram showed that there were 11 and 6 genes identified in the intersection of the dorsal–ventral patterning gene set, respectively, with the highly expressed genes in E13.5 WT dorsal (left) and ventral tongue (right). **(C)** The PPI network was constructed using the 184 *Shh*-related dorsal highly expressed genes, as shown in Supplementary Figure S3D. The violet nodes represent the genes in the *Shh*-related module. **(D)** HC-PIN in CytoCluster extracted the largest and most pivotal module, the *Shh*-related module, from the PPI network. **(E)** ANOVA assay on bulk RNA-seq showed that the transcription of *Shh*, *Foxa2*, *Foxf1*, and *Gsc* was much higher in WT dorsal than in the WT ventral tongues, but it was comparable between the WT ventral and *Osr2-cre* ^*KI*^ *;Rosa26R-Fgf8* ventral tongues. In contrast, *Foxa2* transcription in *Osr2-cre* ^*KI*^ *;Rosa26R-Fgf8* dorsal tongues was significantly weaker than that in WT dorsal tongues, but it was more robust than those in WT ventral and *Osr2-cre* ^*KI*^ *;Rosa26R-Fgf8* ventral tongues. However, the transcription of *Shh*, *Foxf1*, and *Gsc* in *Osr2-cre* ^*KI*^ *;Rosa26R-Fgf8* dorsal tongues showed no significant difference from that in WT dorsal tongues, although it was still more robust than those in WT ventral and *Osr2-cre* ^*KI*^ *;Rosa26R-Fgf8* ventral tongues. **(F)** Immunofluorescence showed that the Foxa2 distribution in the dorsal epithelium and Foxf1 distribution in the lateral–dorsal mesenchyme of E13.5 WT tongue were reduced dorsally in the superficial mesenchyme underlying the dorsal epithelium, but it was still robust in the median mesenchyme of the E13.5 *Osr2-cre* ^*KI*^ *;Rosa26R-Fgf8* tongue. White arrows indicate the Foxa2 and Foxf1 boundary in the superficial dorsal mesenchyme. Similarly, the robust Gsc expression in the dorsal superficial mesenchyme of E13.5 WT tongue was absent in the superficial dorsal mesenchyme of the *Osr2-cre* ^*KI*^ *;Rosa26R-Fgf8* tongue (indicated by red asterisks), while the mild Gsc expression in the medial and ventral mesenchyme of E13.5 WT tongue was slightly impacted in the *Osr2-cre* ^*KI*^ *;Rosa26R-Fgf8* tongue. **(G)** Whole-mount *in situ* hybridization revealed suppressed *Shh* transcription in the E13.5 *Osr2-cre* ^*KI*^ *;Rosa26R-Fgf8* tongue. Scale bar: 200 µm.

Myosin staining showed little discrepancy in the pattern of lingual muscles between WT and *Osr2-Cre* ^*KI*^ *;Rosa26R-Fgf8* mice (Supplementary Figure S2). Thus, we further examined the dorsal–ventral pattern in the *Osr2-Cre* ^*KI*^ *;Rosa26R-Fgf8* tongue via differential gene expression profiling. First, we searched for dorsally and ventrally specific genes in the WT tongue via bulk RNA-seq (Supplementary Figure S3A) and identified 552 dorsal and 504 ventral highly expressed genes, respectively (Figure 2A). A total of 11 and 6 genes were identified from the dorsally and ventrally highly expressed genes, respectively (Figure 2B), through intersection with the established gene set of dorsal–ventral patterning (GO0009953). Notably, both the screened dorsal and ventral highly expressed genes were enriched in the SHH-related signaling pathway (Supplementary Figures S3B, C, E, F). To assess the pivotal role of *Shh*-related genes in dorsal tongue patterning, we extracted the gene sets significantly correlated with *Shh* and *Ptc*, respectively, from the dorsally highly expressed genes (Supplementary Figure S3D). By intersecting the *Shh*-related and *Ptc*-related gene sets, 184 dorsal highly expressed genes were extracted to construct the PPI network (Figure 2C), from which the largest and most pivotal module, the *Shh*-related module, was extracted using the hierarchical clustering algorithm in the protein interaction network (HC-PIN) of CytoCluster (Figure 2D). Interestingly, not only *Shh* and *Ptc* but also *Foxa1*, *Foxa2*, and *Gsc*, all of which were identified from the established gene set for dorsal–ventral patterning, were found in the pivotal *Shh*-related module extracted by HC-PIN, indicating their role in tongue dorsalization. Furthermore, more than five dorsal patterning genes and most genes in the *Shh*-related module were significantly downregulated in the *Osr2-Cre* ^*KI*^ *;Rosa26R-Fgf8* dorsal tongue (Supplementary Figure S3G). Moreover, the *Shh*-related module of the *Osr2-Cre* ^*KI*^ *;Rosa26R-Fgf8* dorsal tongue showed a lower GSVA score than that of the WT counterpart; the GSVA scores of both the WT ventral and *Osr2-Cre* ^*KI*^ *;Rosa26R-Fgf8* ventral tongues were significantly lower than those of the WT dorsal and *Osr2-Cre* ^*KI*^ *;Rosa26R-Fgf8* dorsal tongues; meanwhile, the GSVA score of the *Osr2-Cre* ^*KI*^ *; Rosa26R-Fgf8* ventral tongue did not differ from that of the WT ventral control (Supplementary Figures S3H, I). These data indicated the dorsalizing role of the *Shh*-related module in tongue patterning. ANOVA analyses on bulk RNA-seq showed that *Shh* and *Shh*-related genes (*Foxa2*, *Foxf1*, and *Gsc*) were expressed more robustly in WT dorsal and *Osr2-Cre* ^*KI*^ *;Rosa26R-Fgf8* dorsal tongues than in WT ventral and *Osr2-Cre* ^*KI*^ *;Rosa26R-Fgf8* ventral tongues (Figure 2E). However, only *Foxa2* expression in *Osr2-Cre* ^*KI*^ *;Rosa26R-Fgf8* dorsal tongues was significantly weaker than that in WT dorsal tongues (Figure 2E). Consistent with the ANOVA results, the E13.5 *Osr2-Cre* ^*KI*^ *;Rosa26R-Fgf8* tongues displayed reduced Foxa2 distribution in the lingua dorsal epithelium (Figure 2F). However, although the *Foxf1* and *Gsc* transcription in *Osr2-Cre* ^*KI*^ *;Rosa26R-Fgf8* dorsal tongues was comparable to that in WT dorsal tongues (Figure 2E), their expressing domains were only reduced in the superficial mesenchyme underlying lingual dorsal epithelium but still robust in the medial mesenchyme (Figure 2F). Moreover, whole-mount *in situ* hybridization also indicated a suppressed *Shh* transcription in the *Osr2-Cre* ^*KI*^ *;Rosa26R-Fgf8* lingual dorsal epithelium (Figure 2G). Therefore, the suppressed *Shh*-related dorsal-specific gene expression in *Osr2-Cre* ^*KI*^ *;Rosa26R-Fgf8* tongues indicated an interrupted lingual dorsal–ventral patterning.

### *Fgf8/Fgf18*-related ventral-specific gene expression was extended dorsally in the *Osr2-Cre* ^*KI*^ *;Rosa26R-Fgf8* tongue

**FIGURE 3:**
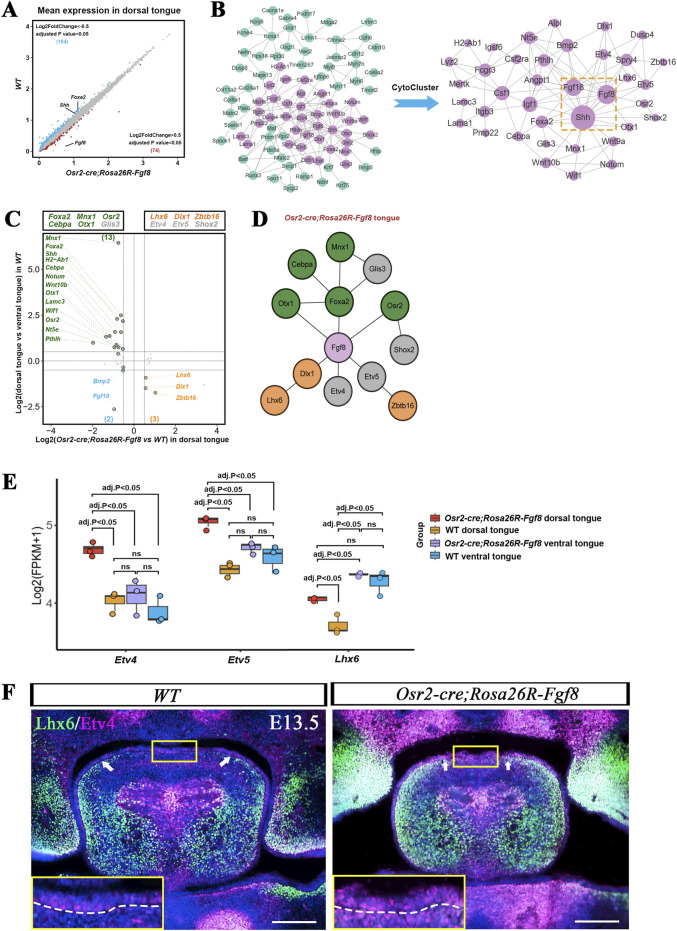
Bulk RNA-seq revealed the *Fgf8/Fgf18-Shh* correlation in dorsal–ventral patterning in E13.5 WT and *Osr2-cre* ^*KI*^ *;Rosa26R-Fgf8* tongues. **(A)** Scatter plot showed 238 DEGs between E13.5 WT dorsal and *Osr2-cre* ^*KI*^ *;Rosa26R-Fgf8* dorsal tongues, among which 74 genes were upregulated (red dots) and 164 genes downregulated (blue dots) in the *Osr2-cre* ^*KI*^ *;Rosa26R-Fgf8* dorsal tongue. **(B)** The PPI network constructed using the 238 DEGs showed the largest and most pivotal module, in which *Fgf8/Fgf18* and *Shh* acted as key nodes, extracted by HC-PIN in CytoCluster. The violet nodes represent the genes in the *Fgf8/Fgf18-Shh* related module. **(C)** Four-quadrant analysis of the *Fgf8/Fgf18-Shh-*related module in E13.5 WT dorsal and *Osr2-cre* ^*KI*^ *;Rosa26R-Fgf8* dorsal tongues. X-axis: E13.5 WT dorsal vs. *Osr2-cre* ^*KI*^ *;Rosa26R-Fgf8* dorsal tongues. Y-axis: E13.5 WT dorsal vs. WT ventral tongues. The genes in the boxes above the four-quadrant plot represent transcription factors. The green-labeled transcription factors were robustly expressed in the WT dorsal tongue, the yellow-labeled transcription factors were upregulated in the *Osr2-cre* ^*KI*^ *;Rosa26R-Fgf8* dorsal tongue, and the blue-labeled transcription factors were robustly expressed in the WT ventral tongue but downregulated in the *Osr2-cre* ^*KI*^ *;Rosa26R-Fgf8* dorsal tongue. The gray transcription factors, namely, *Etv4*, *Etv5*, *Shox2*, and *Glis3*, showed difference between the WT dorsal and ventral tongue but were upregulated or downregulated in the *Osr2-cre* ^*KI*^ *;Rosa26R-Fgf8* dorsal tongue. **(D)** The PPI network of the 12 transcription factors in the boxes above the four-quadrant plots showed that *Fgf8* was the key node connecting *Shh*-related and *Fgf8/Fgf18* modules in the *Osr2-cre* ^*KI*^ *;Rosa26R-Fgf8* tongue. **(E)** Boxplots from bulk RNA-seq showed that the expression of *Etv4* and *Etv5* was upregulated only in the E13.5 *Osr2-cre* ^*KI*^ *;Rosa26R-Fgf8* dorsal tongue but showed little difference among the E13.5 WT dorsal and ventral tongues and the *Osr2-cre* ^*KI*^ *;Rosa26R-Fgf8* ventral tongue. Boxplots also showed that the transcription of *Lhx6* was significantly higher in the E13.5 *Osr2-cre* ^*KI*^ *;Rosa26R-Fgf8* dorsal tongue than in the WT dorsal tongue, but it was comparable in the *Osr2-cre* ^*KI*^ *;Rosa26R-Fgf8* ventral and WT ventral tongues. **(F)** Immunofluorescence showed that the Lhx6 distribution in the lateral–ventral mesenchyme of the E13.5 WT tongue was extended dorsally in the E13.5 *Osr2-cre* ^*KI*^ *;Rosa26R-Fgf8* tongue. White arrows indicate the Lhx6 boundary. Although the robust Etv4 expression in the medial mesenchyme of the E13.5 WT tongue was impacted slightly in the *Osr2-cre* ^*KI*^ *;Rosa26R-Fgf8* tongue, ectopic *Etv4* activation was detected in the dorsal superficial mesenchyme and epithelium of the E13.5 *Osr2-cre* ^*KI*^ *;Rosa26R-Fgf8* tongue. The yellow boxes show the Etv4 distribution in the dorsal superficial mesenchyme and epithelium, which are amplified in the left and lower corners; the dashed lines delineate the boundary between the lingual epithelium and mesenchyme. Scale bar: 200 µm.

We hypothesized that dorsal patterning in the *Osr2-Cre* ^*KI*^ *;Rosa26R-Fgf8* tongue was suppressed by *Fgf8* ectopically activated in the dorsal superficial mesenchyme. By comparing the gene expression profiles of the *Osr2-Cre* ^*KI*^ *;Rosa26R-Fgf8* dorsal to WT dorsal tongues, we constructed a PPI network with 74 upregulated and 164 downregulated DEGs in the *Osr2-Cre* ^*KI*^ *;Rosa26R-Fgf8* dorsal tongue (Figure 3A). The HC-PIN refined from the PPI network by CytoCluster indicated *Shh*, *Fgf8*, and *Fgf18* as hub genes, suggesting suppression of *Shh* by *Fgf8/Fgf18* (Figure 3B). Furthermore, by comparing the DEGs of the WT dorsal to the WT ventral tongue with those of the WT dorsal to the *Osr2-Cre* ^*KI*^ *;Rosa26R-Fgf8* dorsal tongue, we found that the transcription factors *Lhx6*, *Dlx1*, and *Zbtb16*, which are robustly expressed in the ventral WT tongue, were upregulated in the *Osr2-Cre* ^*KI*^ *;Rosa26R-Fgf8* dorsal tongue (the right and lower quadrants in Figure 3C), while the transcription factors, *Foxa2*, *Mnx1*, *Osr2*, *Cebpa1*, and *Otx1*, which were robustly activated in WT dorsal tongue, were downregulated in the *Osr2-Cre* ^*KI*^ *;Rosa26R-Fgf8* dorsal tongue (the left and upper quadrants in Figure 3C). The PPI network constructed using these 12 transcription factors and *Fgf8* indicated that *Fgf8* acted as the key node connecting the dorsally and ventrally specific modules (Figure 3D). However, bulk RNA-seq indicated that although *Etv4/5* transcription in the *Osr2-Cre* ^*KI*^ *;Rosa26R-Fgf8* dorsal tongue was significantly higher than that of the *Osr2-Cre* ^*KI*^ *;Rosa26R-Fgf8* ventral, WT dorsal, and ventral tongues, there was no difference among the *Osr2-Cre* ^*KI*^ *;Rosa26R-Fgf8* ventral, WT dorsal, and ventral tongues (Figure 3E). Moreover, the transcription of *Lhx6*, identified as a ventral marker of the tongue, was significantly higher in the WT ventral tongue than in the WT dorsal tongue but comparable to that in the *Osr2-Cre* ^*KI*^ *;Rosa26R-Fgf8* ventral tongue. Notably, although the *Lhx6* expression in the *Osr2-Cre* ^*KI*^ *;Rosa26R-Fgf8* dorsal tongue was still significantly lower than that in the *Osr2-Cre* ^*KI*^ *;Rosa26R-Fgf8* ventral tongue, it showed no remarkable difference from that in the WT ventral tongue (Figure 3E). Thus, compared to that in the WT dorsal tongue, upregulated *Lhx6* transcription in the *Osr2-Cre* ^*KI*^ *;Rosa26R-Fgf8* dorsal tongue indicated a ventralizing tendency in the *Osr2-Cre* ^*KI*^ *;Rosa26R-Fgf8* dorsal tongue (Figure 3E). Immunofluorescence showed that the *Lhx6-*expressing domain, which is concentrated in the lateral WT ventral tongue, extended dorsally in the *Osr2-Cre* ^*KI*^ *;Rosa26R-Fgf8* tongue (Figure 3F). Coinciding with the bulk RNA-seq consequence, although robust Etv4 staining was detected in the central mesenchyme as in the WT control, the E13.5 *Osr2-Cre* ^*KI*^ *;Rosa26R-Fgf8* tongues also showed an ectopic activation in the dorsal superficial mesenchyme and epithelium (Figure 3F).

On the other hand, the *Osr2-Cre* ^*KI*^ *;Rosa26R-Fgf8* ventral tongue showed no impact of *Fgf8* activation. There were few DEGs when comparing the WT ventral to the *Osr2-Cre* ^*KI*^ *;Rosa26R-Fgf8* ventral tongue (Supplementary Figure S4A). There were 552 highly expressed genes in the WT dorsal tongue compared to the WT ventral tongue and 408 highly expressed genes in the *Osr2-Cre* ^*KI*^ *;Rosa26R-Fgf8* dorsal tongue compared to the *Osr2-Cre* ^*KI*^ *;Rosa26R-Fgf8* ventral tongue, with 314 (56.9%) genes in common (Supplementary Figures S4B, C). In contrast, there were 504 and 526 highly expressed genes in the WT ventral (compared to the WT dorsal tongue) and *Osr2-Cre* ^*KI*^ *;Rosa26R-Fgf8* ventral tongues (compared to the *Osr2-Cre* ^*KI*^ *;Rosa26R-Fgf8* dorsal tongue), respectively, with 356 (70.6%) common genes (Supplementary Figures S4B, D), indicating that *Fgf8* activation significantly impacted dorsalization but had little effect on the ventralization of the tongue.

### FGF8 suppressed *Shh* and *Shh*-related dorsal-specific genes in the embryonic tongue

**FIGURE 4:**
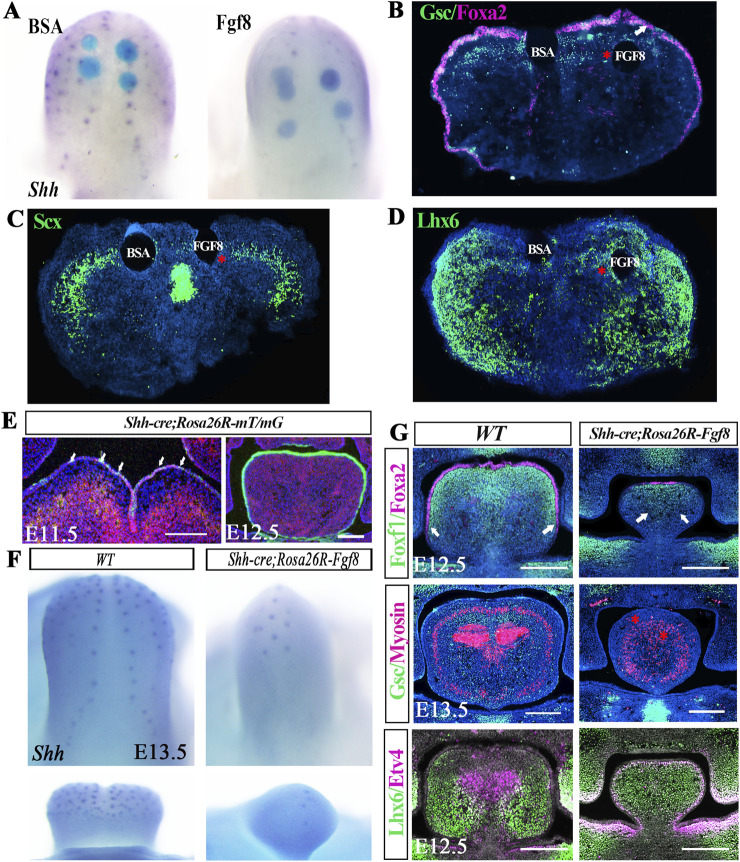
Suppression of *Shh* and *Shh*-related dorsal-specific gene expression by FGF8. **(A)** Whole-mount *in situ* hybridization revealed that the *Shh* transcription in the E13.0 WT tongue was suppressed by implanted FGF8-soaked agarose beads. **(B–D)** Immunofluorescence displayed that implanted FGF8-soaked agarose beads inhibited *Gsc* [red asterisk in **(B)**] and *Scx* expression [red asterisk in **(C)**] but not *Foxa2* expression in the E13.0 WT tongue [white arrow in **(B)**]. In contrast, implanted FGF8-soaked agarose beads induced *Lhx6* expression [red asterisk in **(D)**] in the E13.0 WT tongue. **(E)** *Shh-cre;Rosa26R-mT/mG* tongues exhibited *Cre* activity in the E11.5 (white arrows) and E12.5 lingual epithelium. **(F)** Whole-mount *in situ* hybridization showed significantly reduced *Shh* transcription in the E13.5 *Shh-cre;Rosa26R-Fgf8* tongue. **(G)** Immunofluorescence showed that both Foxf1 (indicated by white arrows) and Foxa2 distribution were reduced to the medial dorsal region in the E12.5 *shh-cre;Rosa26R-Fgf8* tongue; *Gsc* expression was almost reduced in the E13.5 *Shh-cre;Rosa26R-Fgf8* tongue (indicated by red asterisks), while *Lhx6* (indicated by white arrows) and *Etv4* expressions were increased throughout the mesenchyme and epithelium of the E12.5 *Shh-cre;Rosa26R-Fgf8* tongue, respectively. Scale bar: 200 µm.

To examine whether *Shh* and *Shh*-related dorsal gene expressions in the *Osr2-Cre* ^*KI*^ *;Rosa26R-Fgf8* tongue were suppressed by dorsally activated *Fgf8*, FGF8-soaked agarose beads were implanted onto E13.0 WT tongues for 12 h of organ culture. Whole-mount *in situ* hybridization revealed a significant suppression of *Shh* by exogenous FGF8 (Figure 4A; Supplementary Figure S5A; Supplementary Table S1). Immunofluorescence staining indicated that the FGF8-soaked beads suppressed *Gsc* and *Scx* expression (Figures 4B,C; Supplementary Figure S5A; Supplementary Table S1) but induced *Lhx6* expression (Figure 4D; Supplementary Figure S5A; Supplementary Table S1) in the dorsal mesenchyme of WT tongues. More convincingly, when *Fgf8* was activated sporadically in E11.5 and throughout the E12.5 lingual epithelium by *Shh-cre* (Figure 4E), a dramatic decrease in *Shh* transcription was detected in the microglossia of E13.5 *Shh-cre;Rosa26R-Fgf8* mice (Figure 4F; Supplementary Figure S5B; Supplementary Table S2). Consistently, the dorsal-specific expression of *Foxa2*, *Foxf1*, and *Gsc* was remarkably reduced or even completely absent (Figure 4G; Supplementary Figures S5B, C, Supplementary Table S2; Supplementary Table S3), while the ventral-specific gene, *Lhx6*, spread throughout the lingual mesenchyme of the *Shh-cre;Rosa26R-Fgf8* tongue (Figure 4G; Supplementary Figure S5C; Supplementary Table S3). Taken together, FGF8 suppressed the *Shh* and *Shh*-related dorsal-specific gene expression but dorsally extended the *Fgf8/Fgf18*-related ventral-specific genes during tongue development.

### SHH failed to suppress *Fgf18* and *Lhx6* expression in developing tongue

**FIGURE 5:**
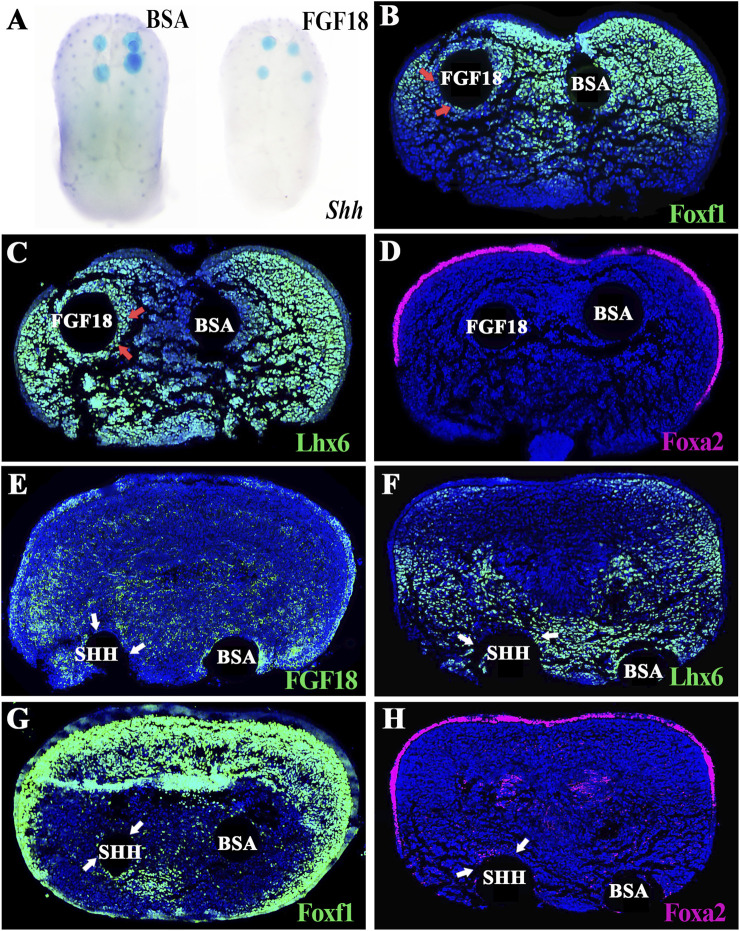
*Ex vivo* culture of E13.0 WT tongues supplemented with FGF18 in the dorsal and SHH in the ventral mesenchyme. **(A)** Whole-mount *in situ* hybridization indicated suppressed *Shh* transcription in the E13.0 WT tongue by implanted FGF18-soaked agarose beads. **(B–D)** Immunofluorescence showed that implanted FGF18-soaked agarose beads inhibited *Foxf1* expression [red arrows in **(B)**] but induced *Lhx6* expression [red arrows in **(C)**]. Meanwhile, *Foxa2* expression in the E13.0 WT tongue was impacted slightly by exogenous FGF18. **(E–H)** In contrast, immunofluorescence showed that the tongues with SHH-soaked agarose beads failed to repress *Fgf18* [white arrows in **(E)**] and *Lhx6* expression [white arrows in **(F)**] in the E13.0 lingual ventral mesenchyme. Similarly, SHH also failed to induce *Foxf1* [white arrows in **(G)**] and *Foxa2* [white arrows in **(H)**] expression in the E13.0 lingual ventral mesenchyme.

To address whether *Fgf18* could suppress *Shh* and *Shh*-related gene expression as *Fgf8* did, FGF18-soaked beads were implanted onto the E13.0 WT tongues for 12 h of organ culture. Similar to the exogenous FGF8, FGF18-soaked beads also inhibited *Shh* expression in the dorsal epithelium (Figure 5A; Supplementary Figure S6A; Supplementary Table S4), reduced the *Foxf1*-expressing domain (Figure 5B; Supplementary Figure S6A; Supplementary Table S4), and induced the *Lhx6*-expressing domain (Figure 5C; Supplementary Figure S6A; Supplementary Table S4) in the dorsal mesenchyme. Notably, FGF18 supplementation had no impact on Foxa2 staining (Figure 5D; Supplementary Figure S6A; Supplementary Table S4), which was identical to the FGF8 supplemented tongue (Figure 4B), indicating that, although initiated by SHH, the maintenance of *Foxa2* expression was independent of SHH. On the other hand, whether *Shh* suppressed *Fgf18* and *Fgf8/18*-related ventral-specific genes was examined by implanting SHH-soaked beads onto E13.0 WT tongue. To our surprise, the SHH-soaked beads neither reduced the *Fgf18-* and *Lhx6-*expressing domains in the ventral mesenchyme (Figures 5E,F; Supplementary Figure S6B; Supplementary Table S5) nor activated *Foxf1* and *Foxa2* expression in the ventral mesenchyme and epithelium (Figures 5G,H; Supplementary Figure S6B; Supplementary Table S5). Therefore, it indicated that although *Fgf8/18* could define lingual ventral patterning by suppressing *Shh* and *Shh*-related dorsal-specific genes, SHH was incapable of dorsalizing the lingual ventral mesenchyme by antagonizing the expression of *Fgf8/18* and *Fgf8/18*-related ventral-specific genes.

## Discussion

In this study, we first showed that a series of dorsal features in mouse embryonic tongues were markedly attenuated by ectopic *Fgf8* activation in the dorsal lingual mesenchyme. Then, we identified dorsally and ventrally specific genes and the key signaling pathways patterning the dorsal–ventral axis of the mouse embryonic tongue by comparing the transcription profiles of the dorsal and ventral tongues. Furthermore, we applied the embryonic tongues from *Osr2-Cre* ^*KI*^ *;Rosa26R-Fgf8* and *Shh-Cre;Rosa26R-Fgf8* mice to address the critical role of *Fgf8/18* in lingual ventral patterning by antagonizing dorsal *Shh* and *Shh*-related genes. In summary, our study not only confirmed the existence of dorsal–ventral patterning in the embryonic tongue but also identified the factors involved in this patterning, providing deeper insights into the cellular and molecular mechanisms underlying tongue development and offering clues for interpreting the clinical manifestations of lingual deformities.

A previous study indicated that in the early mandibular arch, oral FGF8-induced *Lhx6* expression antagonized the extension of aboral endothelin-induced *Gsc* expression to establish oral–aboral patterning (Tucker et al., 1999). During the early stage of tongue development, the paired lateral lingual swellings emerge from the oral mesenchyme, grow rapidly toward the midline of the mandibular arch, and ultimately fuse into the anterior two-thirds of the tongue (Cobourne et al., 2019). Thus, the *Lhx6* expression in ventral lingual mesenchyme was intensively assumed to have originated from the oral mesenchyme and maintained by *Fgf8* activated in E10.5 lingual epithelium (Jung et al., 1999), which was supported by the dorsal extension of *Lhx6* in the *Osr2-Cre* ^*KI*^ *;Rosa26R-Fgf8* and *Shh-Cre;Rosa26R-Fgf8* tongues and the induced *Lhx6* expression in the WT tongue by exogenous FGF8. During tongue development, *Fgf8* expression was reduced at E11.5 and absent at E13.5, followed by the robust *Fgf18* transcription in the ventral mesenchyme at E12.5 (Jung et al., 1999; Du et al., 2016). Belonging to the FGF8 subfamily, FGF8 and FGF18 have been shown to share similar receptor affinities downstream signaling pathways, and target genes (Jiang et al., 2013; Marashi et al., 2019). Thus, we proposed that *Fgf18* succeeded *Fgf8* to maintain *Lhx6* expression in the embryonic tongue, which is supported by *Lhx6* expression in WT dorsal lingual mesenchyme induced by exogenous FGF18.

Our study also showed the diminished *Shh* and *Shh*-related dorsal-specific genes in the dorsal mesenchyme of *Osr2-Cre* ^*KI*^ *;Rosa26R-Fgf8* and *Shh-Cre;Rosa26R-Fgf8* tongues, along with the reduced *Shh* and *Foxf1* expression in the WT tongue supplemented with FGF8/18. These results further indicated that FGF8/18 defined lingual ventral patterning by antagonizing *Shh* expression. Thus, the mutual interaction between *Shh* and *Fgf8/18* in tongue development requires elucidation. A recent study proposed the *Shh-Foxf1/2-Fgf18-Shh* circuit during palatogenesis, in which ectopic *Fgf18* activation in the palatal mesenchyme and *Shh* inactivation in the palatal epithelium were detected in *Wnt1-cre;Foxf1* ^*f/f*^ *;Foxf2* ^*f/f*^ mice, along with repression of *Shh* transcription in the palatal epithelium caused by exogenous FGF18 (Xu et al., 2016). Further investigation indicated the direct suppression of *Fgf18* expression by Foxf2 in the palatal mesenchyme (Xu et al., 2020). Combined with the complementary pattern of *Shh* and *Fgf8* in the early facial primordia (Abzhanov et al., 2007; Marchini et al., 2025) and suppressed Shh transcription by ectopic Fgf8 activation in the palatal mesenchyme (Wu et al., 2015), all these studies indicated an antagonization of *Shh* by *Fgf8/18* in lingual dorsal–ventral patterning.

In contrast to FGF8/18 in ventral patterning, the DEGs that were highly expressed in WT dorsal tongue indicated the critical role of *Shh* and *Shh*-related dorsal-specific genes in lingual dorsalization. A recent study showed a complementary pattern of SHH and BMP4 signaling along the oral–aboral axis during early mandibular patterning and indicated that SHH signaling specified the oral fate and the oral–aboral pattern of the mandibular mesenchyme through Foxf1/2. Inactivation of SHH signaling in CNCCs led to aglossia, accompanied by disrupted oral–aboral patterning of the mandibular bone due to expanded BMP signaling activity (Xu et al., 2019). These findings indicated that the mutual antagonization between *Shh-Foxf1/2* and *Fgf8/18-Lhx6* defined dorsal–ventral patterning during early lingual development. However, the exogenous SHH appeared to be incapable of activating *Shh*-related dorsal-specific genes or suppressing *Lhx6* transcription in WT lingual ventral mesenchyme, indicating a specific capacity of responding to SHH in the lingual dorsal mesenchyme. There is the other possibility that the lingual dorsal mesenchyme is not derived from the *Lhx6+* oral mesenchyme but immigrates into the lingual primordium from the second or third branchial arch. A recent study showed that the SHH-Wnt5a-induced invasion of a CXCL12+ mesenchyme into the lingual primordium was indispensable for lingual morphogenesis, implicating the heterogeneity of the lingual mesenchyme (Zhang et al., 2022). Moreover, inactivation of *Shh* or SHH signaling resulted in aglossia or hypoglossia (Liu et al., 2004; Billmyre and Klingensmith, 2015; Millington et al., 2017; Huang et al., 2023), indicating that the role of the dorsal mesenchyme is not limited to only patterning in tongue development. Therefore, a transcriptomic atlas in single-cell resolution is required to address the cellular diversity and the distinct roles of multiple lingual mesenchymal populations during tongue development. Additionally, the *in vivo* model overexpressing *Fgf18* by *Osr2-cre* ^*KI*^ is required to confirm its influence on *Shh* transcription and tongue development. Finally, our study only focused on the dorsal–ventral patterning of the anterior two-thirds of the tongue; however, defining the dorsal–ventral patterning process in the posterior one-third of the tongue still requires further exploration because of the invasion of the external lingual muscles, especially the genioglossus.

## Data Availability

The datasets presented in this study can be found in online repositories. The names of the repository/repositories and accession number(s) can be found at: https://www.ncbi.nlm.nih.gov/, PRJNA1278902.
