# Supplementary material for: Fgf8/18 antagonizes Shh expression in lingual ventral–dorsal patterning
Source: Front Cell Dev Biol. 2026 Feb 10;14:1724475. doi: 10.3389/fcell.2026.1724475 (PMC12929486; doi:10.3389/fcell.2026.1724475)
Supplement: Supplementary file 1 [file DataSheet2.pdf]

Supplementary Table 1. Corrected fluorescence/color intensity with exogenous FGF8

	BSA		FGF8		
	Mean	SD	Mean	SD	P value
Shh	19.65	4.20	10.63	1.54	0.025017
Gsc	11.03	0.62	5.50	0.43	0.000217
Foxa2	24.41	2.47	23.07	1.88	0.496061
Scx	11.85	1.81	2.97	0.99	0.001725
Lhx6	5.22	0.65	19.42	1.57	0.000133

Supplementary Table 2. The area percentages/corrected color intensity of positive signals in lingual epithelium

	WT		*Shh-Cre;Rosa26R-Fgf8*		
	Mean	SD	Mean	SD	P value
Shh	25.99	2.55	6.17	2.17	0.000509
Foxa2	71.24	4.03	22.87	1.95	0.000048
Etv4	0.71	0.17	0.68	0.44	0.913734

Supplementary Table 3. The percentage of positive signal areas in lingual mesenchyme

	WT		*Shh-Cre;Rosa26R-Fgf8*		
	Mean	SD	Mean	SD	P value
Foxf1	51.04	2.57	4.55	1.17	0.000009
Gsc	6.12	0.67	0.19	0.06	0.000106
Myosin	8.99	1.31	7.45	1.60	0.266876
Lhx6	19.46	2.56	32.66	5.05	0.015628
Etv4	13.56	2.11	12.90	1.16	0.660987

Supplementary Table 4. Corrected fluorescence/color intensity with exogenous FGF18

	BSA		FGF18		
	Mean	SD	Mean	SD	P value
Shh	23.83	2.74	8.32	1.85	0.001252
Foxf1	38.82	1.70	15.21	2.50	0.000172
Lhx6	3.75	0.44	20.51	2.36	0.000268
Foxa2	21.40	1.54	23.91	3.67	0.336436

Supplementary Table 5. Corrected fluorescence/color intensity with exogenous SHH

	BSA		SHH		
	Mean	SD	Mean	SD	P value
Fgf18	15.12	1.31	15.72	2.94	0.762931
Lhx6	13.80	0.96	12.90	2.03	0.527394
Foxf1	1.59	0.40	0.93	0.18	0.059858
Foxa2	0.06	0.10	0.22	0.25	0.351905
