# Supplementary material for: Fgf8/18 antagonizes Shh expression in lingual ventral–dorsal patterning
Source: Front Cell Dev Biol. 2026 Feb 10;14:1724475. doi: 10.3389/fcell.2026.1724475 (PMC12929486; doi:10.3389/fcell.2026.1724475)
Supplement: Supplementary file 2 [file DataSheet1.pdf]

Supplementary Figure 1. The tongue deformity in *Osr2-cre*^{KI}*;Rosa26R-Fgf8* mouse embryos. (**A**) The tongues in *Osr2-cre*^{KI}*;Rosa26R-Fgf8* mouse embryos were pushed out of oral cavity (indicated by the white arrowheads) because of micrognathia. (**B**) Statistical assay of the width and length of *Osr2-cre*^{KI}*;Rosa26R-Fgf8* tongues. *: $p<0.05$; **: $p<0.01$; ***: $p<0.001$. (**C**) The cross views of E13.5 and E16.5 *Osr2-cre*^{KI}*;Rosa26R-Fgf8* heads showed the deformity in posterior tongue resulted from the compression by enlarged palatal shelves. However, Myosin staining in E13.5 *Osr2-cre*^{KI}*;Rosa26R-Fgf8* tongue showed little impacts on intrinsic and extrinsic muscle pattern, except the slightly decreased Myosin staining in longitudinal muscles (indicated by black arrowheads). (**D**) TUNEL assay showed little alteration on cell apoptosis in *Osr2-cre*^{KI}*;Rosa26R-Fgf8* tongue. Scale bar: 200 µm.

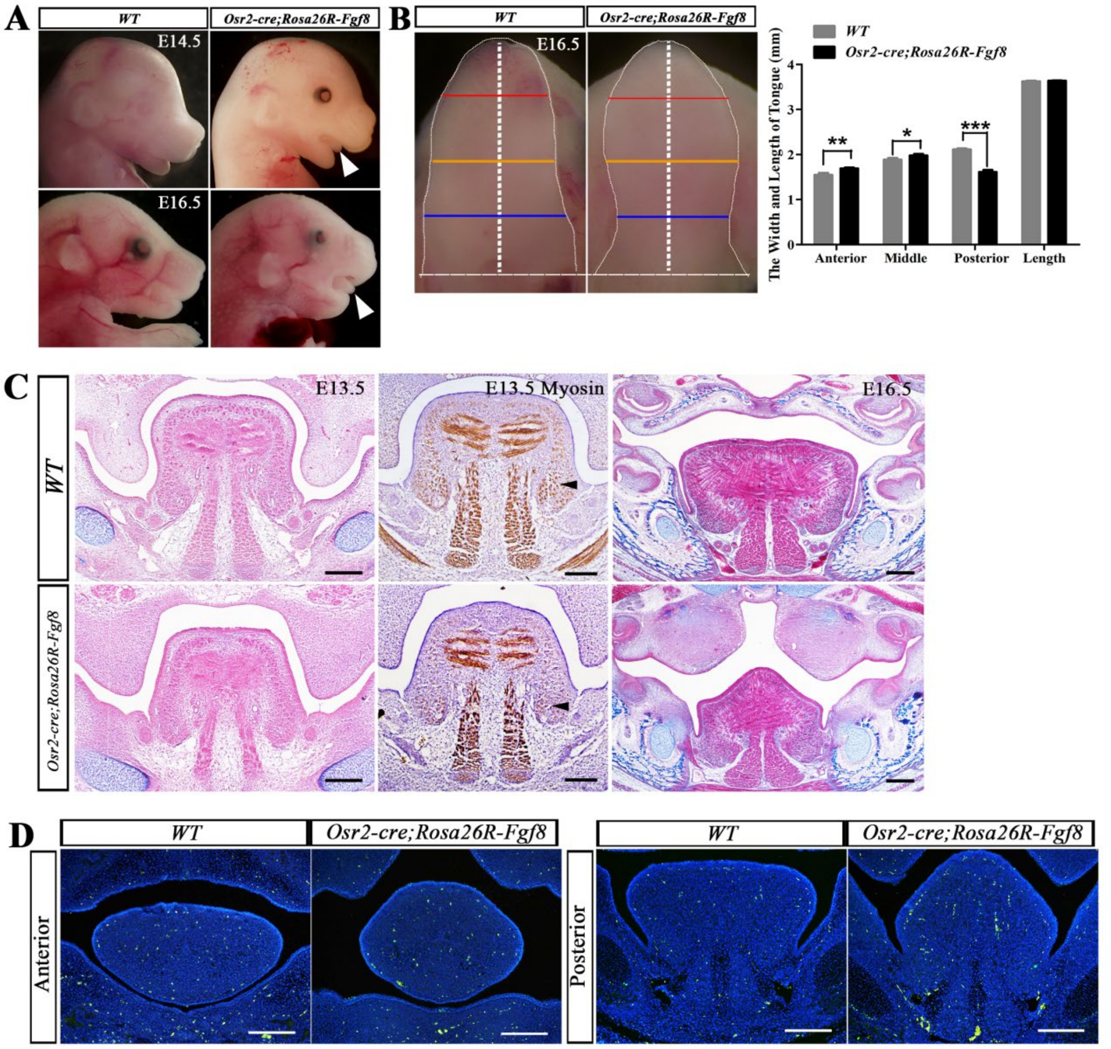

Figure. S1.

Supplementary Figure 2. Muscle patterning in *Osr2-cre^{KI};Rosa26R-Fgf8* tongue was little impacted. (**A-A'''**) Masson staining of E14.5 WT anterior tongue. (**B-B'''**) Masson staining of E14.5 *Osr2-cre^{KI};Rosa26R-Fgf8* anterior tongue. (**C-C'''**) Immunohistochemical staining of Myosin in E14.5 WT anterior tongue. (**D-D'''**) Immunohistochemical staining of Myosin in E14.5 *Osr2-cre^{KI};Rosa26R-Fgf8* anterior tongue. (**E-E'''**) Masson staining of E16.5 WT anterior tongue. (**F-F'''**) Masson staining of E16.5 *Osr2-cre^{KI};Rosa26R-Fgf8* anterior tongue. (**G-G'''**) Immunohistochemical staining of Myosin in E16.5 WT anterior tongue. (**H-H'''**) Immunohistochemical staining of Myosin in E16.5 *Osr2-cre^{KI};Rosa26R-Fgf8* anterior tongue. (**I-I'''**) Masson staining of E14.5 WT posterior tongue. (**J-J'''**) Masson staining of E14.5 *Osr2-cre^{KI};Rosa26R-Fgf8* posterior tongue. (**K-K'''**) Immunohistochemical staining of Myosin in E14.5 WTmposterior tongue. (**L-L'''**) Immunohistochemical staining of Myosin in E14.5 *Osr2-cre^{KI};Rosa26R-Fgf8* posterior tongue. (**M-M'''**) Masson staining of E16.5 WT posterior tongue. (**N-N'''**) Masson staining of E16.5 *Osr2-cre^{KI};Rosa26R-Fgf8* posterior tongue. (**O-O'''**) Immunohistochemical staining of Myosin in E16.5 WT posterior tongue. (**P-P'''**) Immunohistochemical staining of Myosin in E16.5 *Osr2-cre^{KI};Rosa26R-Fgf8* posterior tongue. SLM: superior longitudinal muscle; ILM: inferior longitudinal muscle; TM: transverse muscle; VM: vertical muscle; the images in SLM, ILM and TM&VM rows represent the amplified views in the blue, red and yellow boxes of the corresponding Anterior or Posterior rows; black asterisks: the superficial mesenchyme in WT tongues; red asterisks: the superficial mesenchyme in *Osr2-cre^{KI}; Rosa26R-Fgf8* tongues; Scale bar: 200 μm.

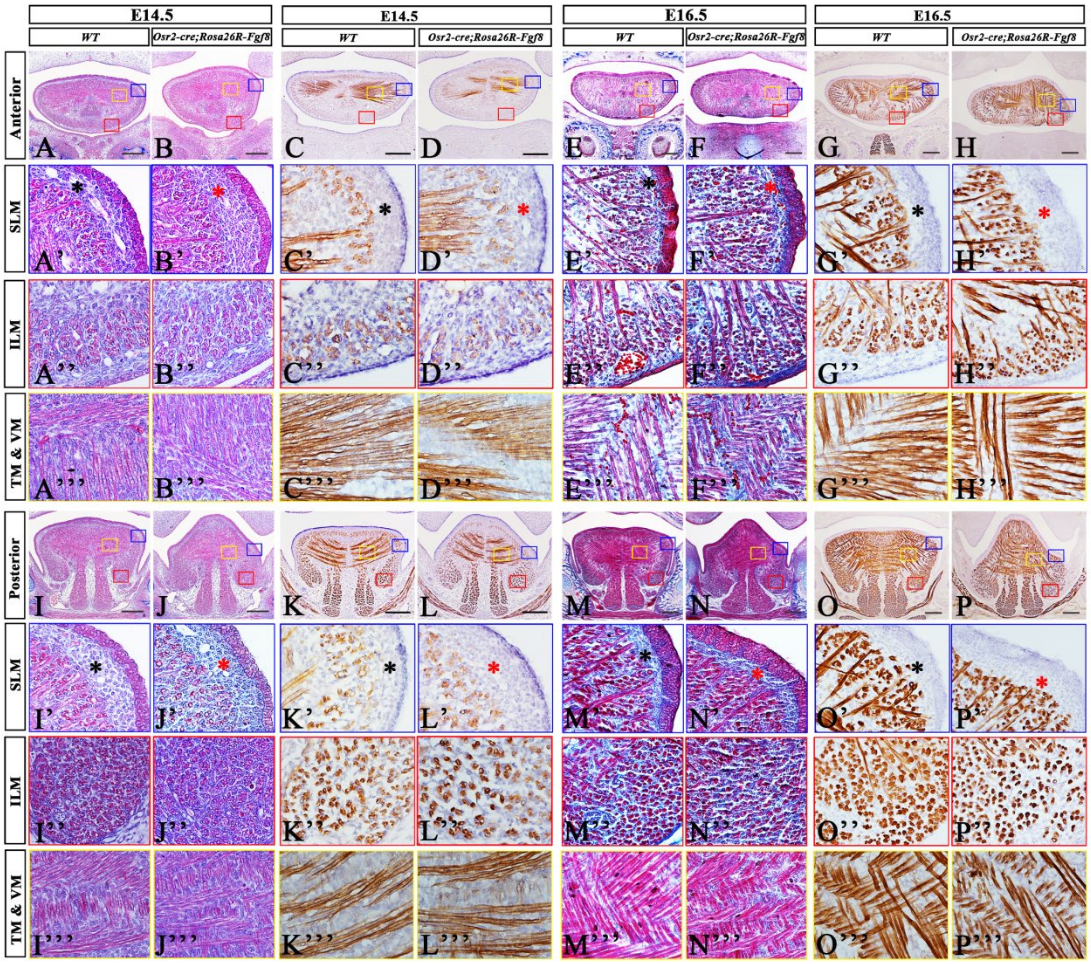
E14.5
E14.5
E16.5
E16.5
WT
Osr2-cre;Rosa26R-Fgf8
Anterior
SLM
ILM
TM & VM
Posterior
SLM
ILM
TM & VM

Figure. S2.

Supplementary Figure 3. The expression of *Shh*-related genes in E13.5 WT dorsal and *Osr2-cre*^{KI}*;Rosa26R-Fgf8* dorsal tongues. (**A**) The collection of the dorsal and ventral parts in E13.5 WT and *Osr2-cre*^{KI}*; Rosa26R-Fgf8* tongues, and the flowchart of bulk RNA-Seq data analysis. (**B**) Correlation between *Shh* and *Ptch1* expression levels from bulk RNA-Seq of E13.5 WT dorsal and ventral tongues. (**C**) Horizontal bar plot showed that there were 370 and 220 highly expressed genes identified via bulk RNA-Seq in E13.5 WT dorsal tongues that are correlated (correlation cofficient > 0.9) with *Shh* and *Ptch1*, respectively. Bar length represents gene count. (**D**) Venn diagram showed that there were 184 highly expressed genes correlated with both *Shh* and *Ptch1* (correlation coefficient > 0.9) in E13.5 WT dorsal tongues. (**E**) Enrichment for canonical pathways showed that the 11 dorsal-ventral patterning related genes in E13.5 WT dorsal tongue were enriched for SHH-related signaling. (**F**) The 6 dorsal-ventral patterning related genes in E13.5 WT ventral tongue were also enriched for SHH-related signaling. (**G**) Horizontal bar plot showed the expression changes of the identified genes in **Figure 2F** by comparing E13.5 WT dorsal tongue to *Osr2-cre*^{KI}*;Rosa26R-Fgf8* dorsal tongues. (**H**) GSVA showed the reduced enrichment of the tongue dorsal geneset (comprising all genes from **Figure 2D** by comparing E13.5 WT dorsal tongue to *Osr2-cre*^{KI}*;Rosa26R-Fgf8* dorsal tongue). (**I**) Horizontal bar plot showed that by comparing E13.5 WT dorsal tongue to *Osr2-cre*^{KI}*;Rosa26R-Fgf8* dorsal tongue, the expression of five key genes in **Figure 2D** was decreased.

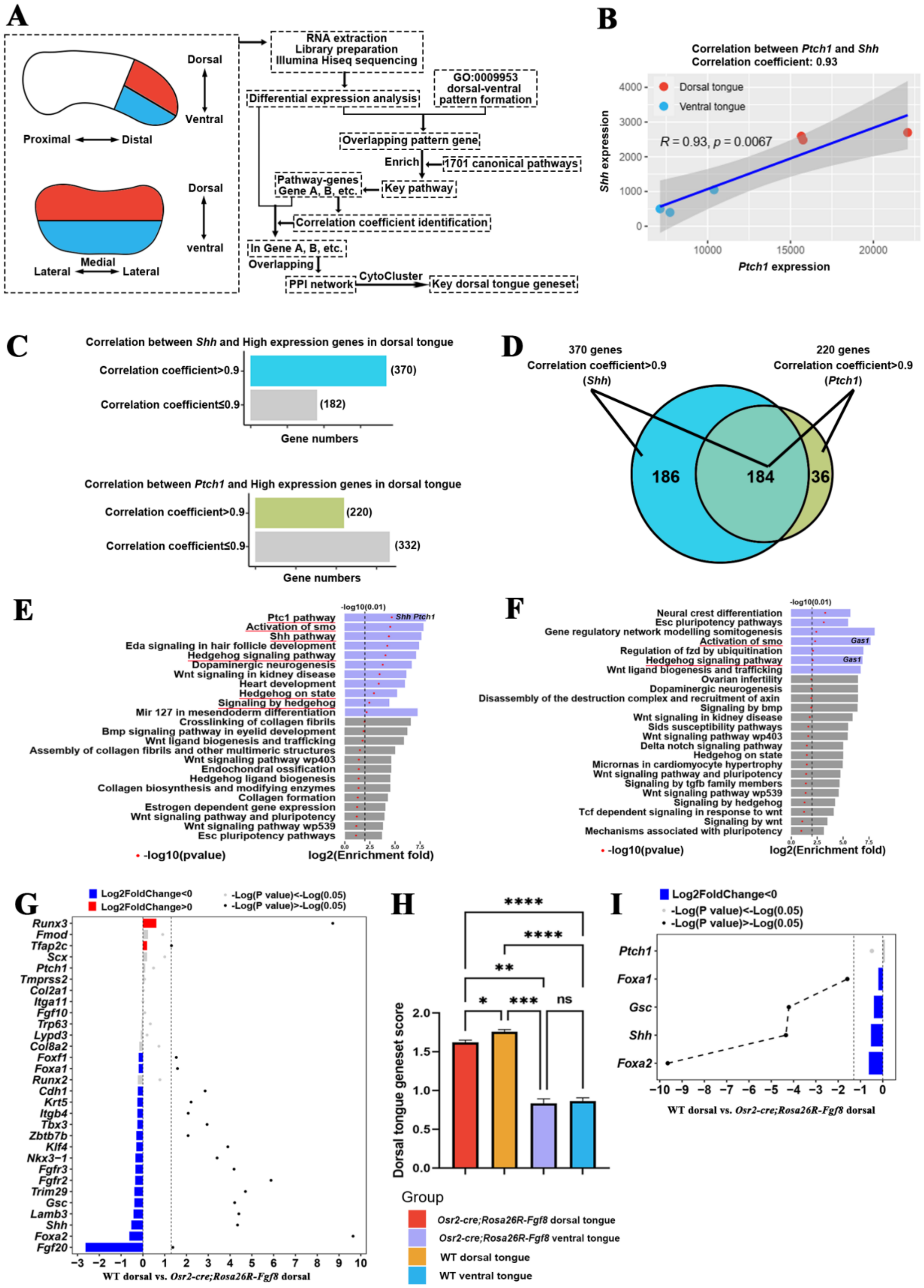

Figure. S3.

Supplementary Figure 4. The DEGs in the dorsal and ventral portions of E13.5 WT and *Osr2-cre; Rosa26R-Fgf8* tongues. (**A**) Scatter plots displayed no DEG by comparing the gene expression profile between E13.5 ventral WT and *Osr2-cre*^{KI}*; Rosa26R-Fgf8* tongues. (**B**) Bar plots showed that in E13.5 WT tongue, there were 552 genes expressed higher in WT dorsal tongue than in WT ventral tongue, while 504 genes expressed higher in WT ventral ventral tongue than in WT dorsal tongue; in E13.5 *Osr2-cre*^{KI}*;Rosa26R-Fgf8* tongue, there were 408 genes expressed in dorsal tongue higher than those in ventral tongue, 526 genes expressed in the ventral tongue higher than those in dorsal tongue. (**C**) Venn diagram showed the intersection of the DEGs higly expressed in both E13.5 WT dorsal tongue and *Osr2-cre*^{KI}*; Rosa26R-Fgf8* dorsal tongues. (**D**) Venn diagram showed the intersection of the DEGs highly expressed in both E13.5 WT ventral and *Osr2-cre*^{KI}*;Rosa26R-Fgf8* ventral tongue.

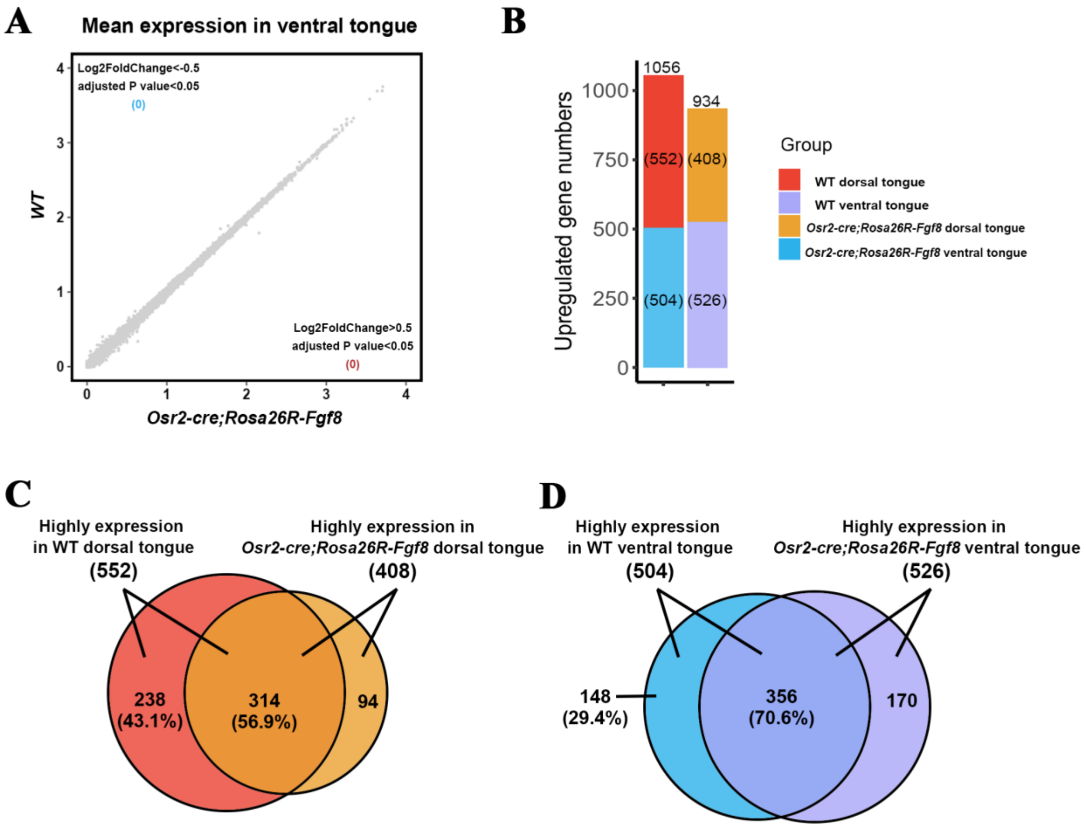

Figure. S4.

Supplementary Figure 5. The quantified gene expression in E13.5 WT tongues with exogenous FGF8 and *Shh-cre;Rosa26R-Fgf8* tongues. (**A**) Corrected color/ fluorescence intensity of *Shh*, Gsc, Foxa2, Scx and Lhx6 with exogenous FGF8 supplement in E13.5 WT tongues. (**B**) The corrected color intensity/area percentages of *Shh*, Foxa2 and Etv4 in E13.5 WT and *Shh-cre;Rosa26R-Fgf8* lingual epithelium. (**C**) The area percentages of Foxf1, Gsc, Myosin, Lhx6 and Etv4 in E13.5 WT and *Shh-cre;Rosa26R-Fgf8* lingual mesenchyme.

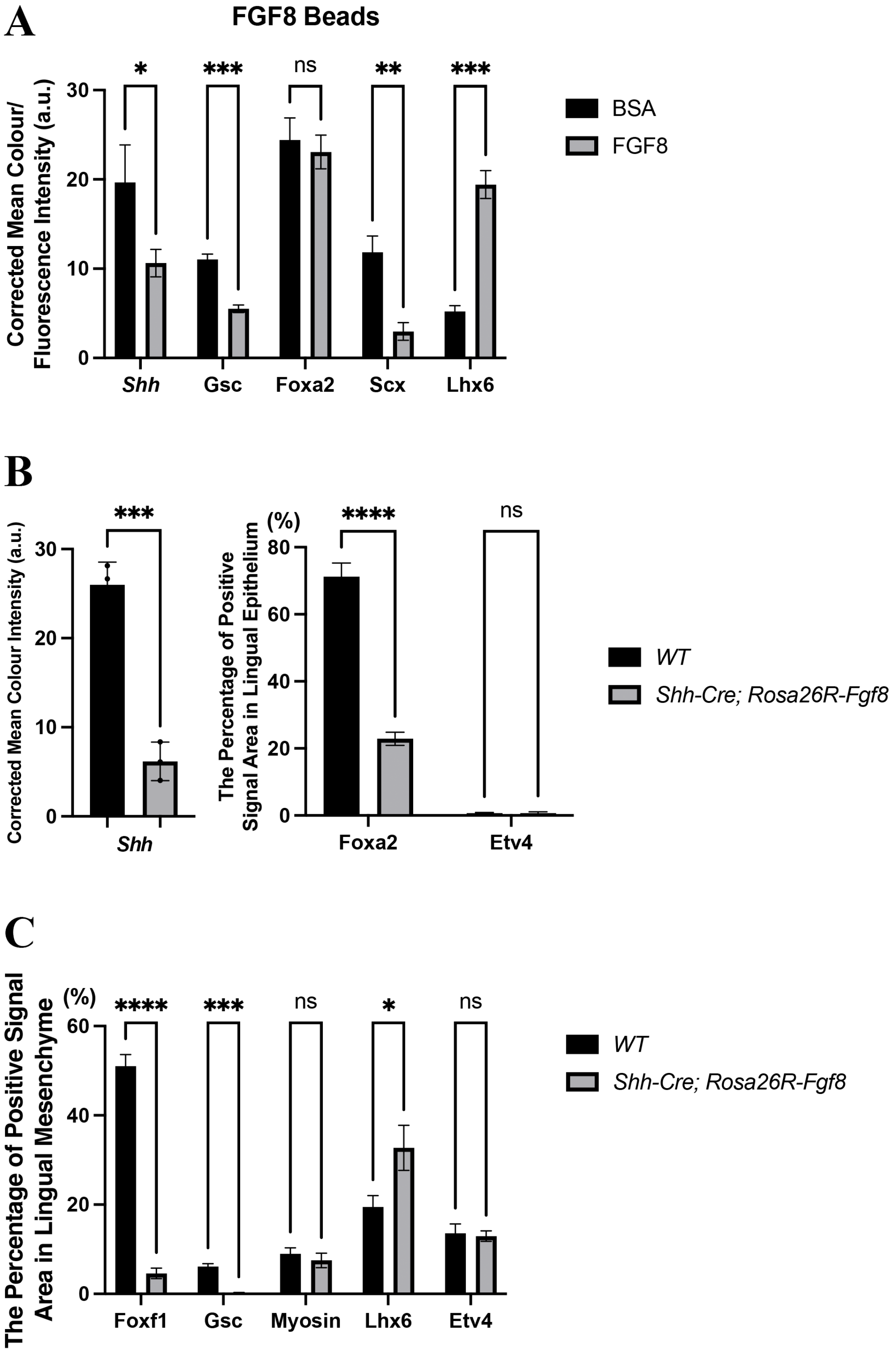
A
FGF8 Beads
Corrected Mean Colour/ Fluorescence Intensity (a.u.)
*
***
ns
**
***
Shh
Gsc
Foxa2
Scx
Lhx6
BSA
FGF8
B
Corrected Mean Colour Intensity (a.u.)
***
Shh
The Percentage of Positive Signal Area in Lingual Epithelium
(%)
****
ns
Foxa2
Etv4
WT
Shh-Cre; Rosa26R-Fgf8
C
The Percentage of Positive Signal Area in Lingual Mesenchyme
(%)
****
***
ns
*
ns
Foxf1
Gsc
Myosin
Lhx6
Etv4
WT
Shh-Cre; Rosa26R-Fgf8

Figure. S5.

Supplementary Figure 6. The quantified gene expression in E13.5 WT tongues supplemented with exogenous FGF18 or SHH. (**A**) Corrected color/ fluorescence intensity of *Shh*, Foxf1, Lhx6 and Foxa2 with exogenous FGF18. (**B**) Corrected fluorescence intensity of Fgf18, Lhx6, Foxf1 and Foxa2 with exogenous SHH.

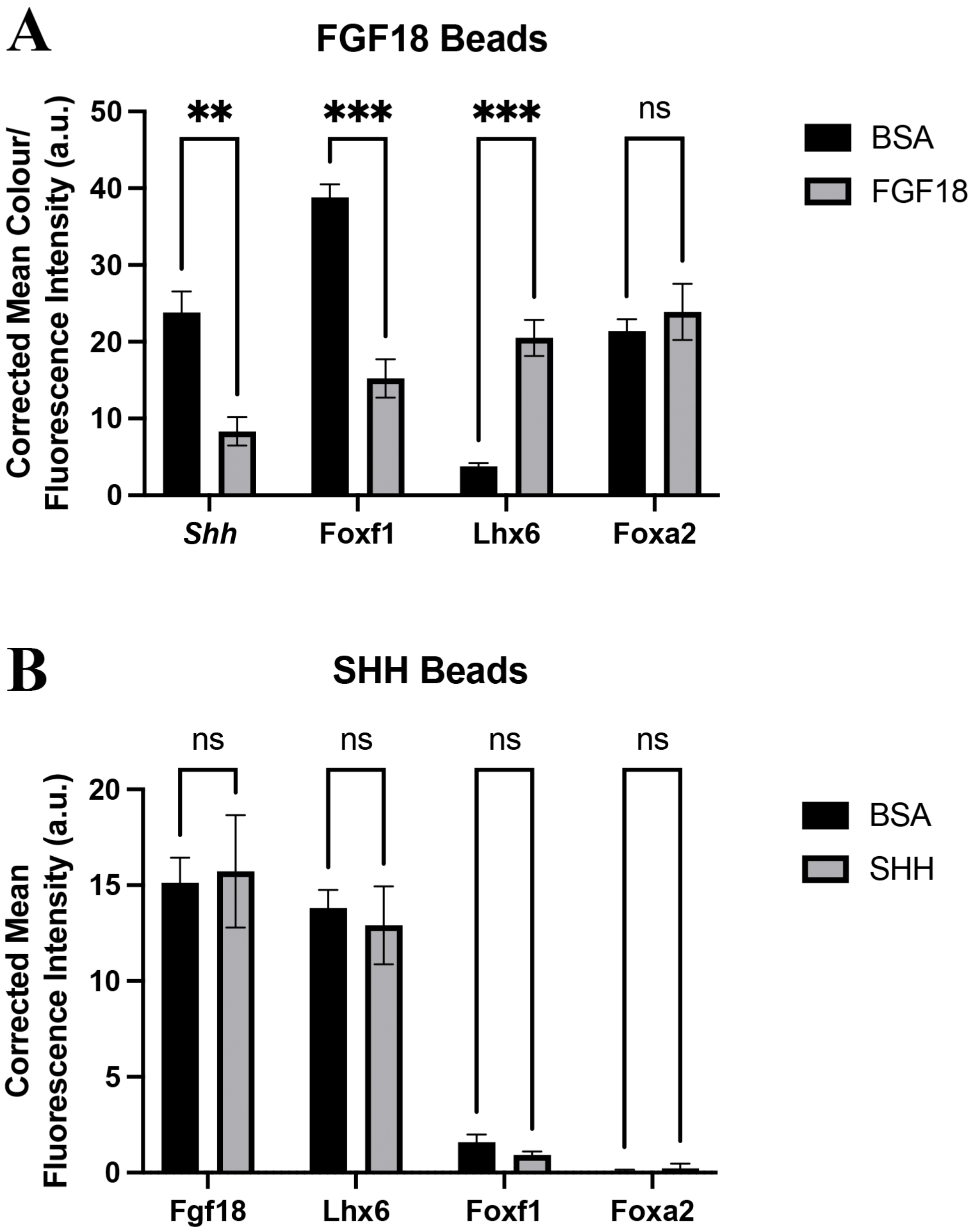

Figure. S6.
